# Microfluidic Detection Platform for Determination of Ractopamine in Food

**DOI:** 10.3390/bios14100462

**Published:** 2024-09-26

**Authors:** Cheng-Xue Yu, Kuan-Hsun Huang, To-Lin Chen, Chan-Chiung Liu, Lung-Ming Fu

**Affiliations:** 1 Department of Engineering Science, National Cheng Kung University, Tainan 70101, Taiwan; n98121509@gs.ncku.edu.tw (C.-X.Y.); n96104496@gs.ncku.edu.tw (K.-H.H.); n98134049@gs.ncku.edu.tw (T.-L.C.); 2 Department of Food Science, National Pingtung University of Science and Technology, Pingtung 91201, Taiwan; ccliu@mail.npust.edu.tw

**Keywords:** ractopamine, microfluidic, gold nanoparticles, micro-spectrophotometer

## Abstract

A novel microfluidic ractopamine (RAC) detection platform consisting of a microfluidic RAC chip and a smart analysis device is proposed for the determination of RAC concentration in meat samples. This technology utilizes gold nanoparticles (AuNPs) modified with glutamic acid (GLU) and polyethyleneimine (PEI) to measure RAC concentration in food products. When RAC is present, AuNPs aggregate through hydrogen bonding, causing noticeable changes in their optical properties, which are detected using a self-built UV–visible micro-spectrophotometer. Within the range of 5 to 80 ppb, a linear relationship exists between the absorbance ratio (A_693nm_/A_518nm_) (Y) and RAC concentration (X), expressed as Y = 0.0054X + 0.4690, with a high coefficient of determination (R^2^ = 0.9943). This method exhibits a detection limit of 1.0 ppb and achieves results within 3 min. The practical utility of this microfluidic assay is exemplified through the evaluation of RAC concentrations in 50 commercially available meat samples. The variance between concentrations measured using this platform and those determined via liquid chromatography–tandem mass spectrometry (LC-MS/MS) is less than 8.33%. These results underscore the viability of the microfluidic detection platform as a rapid and cost-effective solution for ensuring food safety and regulatory compliance within the livestock industry.

## 1. Introduction

Ractopamine (RAC) is a β-agonist that enhances protein synthesis in muscles, leading to increased rates of weight gain, feeding efficiency, and muscle mass in livestock, particularly cattle and swine [1]. However, food additives such as RAC can easily be ingested by humans through residues in meat products, and high levels of these additives may cause acute poisoning of the cardiovascular, nervous, and respiratory systems [2,3]. Therefore, the Joint FAO/WHO Expert Committee on Food Additives stipulates that the daily intake of RAC should not exceed 1 μg/kg body weight per day. Additionally, some countries impose restrictions on the use of RAC in animal husbandry. For instance, meat products from animals treated with RAC may be prohibited from entering markets where RAC is banned. Effective methods for determining RAC concentrations in food products are therefore essential for both health and trade reasons.

Various methods are available for detecting RAC, including surface-enhanced Raman scattering (SERS) [4], electrochemical detection [5,6,7,8,9], high-performance liquid chromatography (HPLC) [10], LC-MS/MS [11,12,13], capillary electrophoresis [14,15], and immunoassays [16,17,18,19]. However, all these methods are time-consuming and require expensive and bulky instruments and skilled professionals. Therefore, there is an urgent need for low-cost, accurate, and real-time methods for RAC detection.

Microfluidics, which integrates the disciplines of chemistry, physics, biotechnology, and medicine, finds extensive applications in automated and rapid diagnostics [20,21,22,23,24,25]. Compared to conventional large-scale analysis systems, lab-on-a-chip (LOC) microfluidic devices are capable of performing multiple biochemical analysis procedures on a single chip, providing many practical advantages, including reduced sample and reagent volumes, straightforward operation, lower manufacturing costs, and enhanced portability [26,27,28,29,30].

In recent years, microfluidic-based systems for food safety detection have developed rapidly [31,32,33,34,35]. For example, Liu et al. [36] introduced a light-shielding reactive PMMA/paper microfluidic detection platform for the determination of cyclamate concentration in commercial foods. Chen et al. [37] presented a microfluidic analytical detection system including a microfluidic PMMA paper chip and a smart analysis device to determine the content of sodium dehydroacetate in food. Ko et al. [38] developed an innovative assay platform comprising a finger pump microchip and a Wi-Fi-based analysis system to detect the concentration of methylparaben levels in foods. The platform showed a reliable measurement performance over the methylparaben concentration range of 100 to 3000 ppm. Moreover, the measurement results deviated from those obtained using a conventional large-scale HPLC technique by no more than 5.88%.

Gold nanoparticles (AuNPs) have attracted significant attention for biomedicine and biomonitoring applications in recent years due to their surface plasmon resonance and excellent optical properties [39,40,41,42]. However, while AuNPs have high stability during long-term storage, they can still aggregate when other salts or buffers are introduced. It has been shown that this problem can be overcome by modifying the properties of AuNPs through the addition of specific modifiers, such as -NH, -OH, and -COOH groups [43,44,45,46,47]. These modifiers not only enhance the stability of the AuNPs but also improve their biocompatibility and hydrophobicity, thereby extending their applicability to areas such as targeted drug delivery, diagnostic assays, and environmental sensing.

The functionalization of AuNPs for the detection of RAC can be achieved by exploiting the amine (-NH) groups of polyethyleneimine (PEI) and glutamic acid (GLU). PEI modifies the surface charge of the AuNPs, which promotes their stable dispersion through electrostatic repulsion and imparts to them a red color. GLU then forms hydrogen bonds with the -OH groups on the RAC using its own -C=O functionality, resulting in AuNP aggregation and a color shift from red to blue [48]. PEI is known for its high positive charge density and proton sponge effect, which allows it to efficiently condense nucleic acids or interact with negatively charged surfaces. In the context of nanoparticle synthesis or functionalization, PEI can promote the binding of biomolecules or enhance the stability of nanoparticles through electrostatic interactions. The reason for choosing GLU is that GLU is abundant and easily available in biological systems, and its chemical structure and side chain characteristics can provide favorable interactions with RAC molecules. The side chain of GLU contains carboxyl groups, which can participate in hydrogen bonding with RAC. This interaction may increase the specificity and efficiency of RAC capture.

The present study introduces a microfluidic system for detecting RAC in food samples, which integrates a microfluidic RAC chip with a smart analysis device. In the detection process, 80 µL of an RAC sample is added, and then the pH buffer and GLU-PEI-AuNPs are injected into the reaction chamber using syringe pumps. The reactants are passed through a serpentine mixing channel and enter the detection zone of the chip. The microfluidic RAC chip is subsequently inserted into the smart analysis device, where the absorbance of the reactant at wavelengths of 693 nm and 518 nm is measured using a micro-spectrophotometer. Finally, the RAC concentration is extracted from the photometer signal using a previously prepared calibration curve. The entire reaction and analysis process only takes only 3 min to obtain results, and the designed platform is low-cost and miniaturized, enabling instant on-site measurement of RAC concentration.

## 2. Materials and Methods

### 2.1. Fabrication of Microfluidic RAC Chip

**Figure 1:**
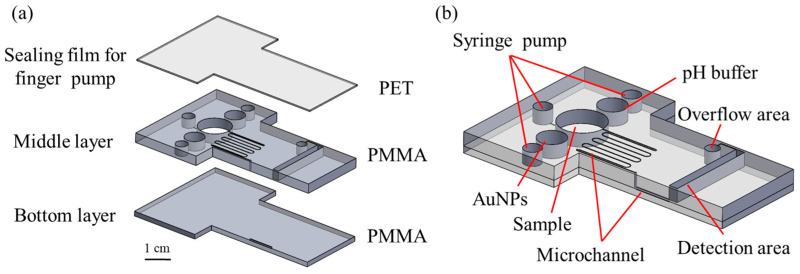
Schematic illustrations showing (**a**) the multi-layer structure of the microfluidic RAC chip and (**b**) the assembled chip.

Figure 1a presents the basic configuration and composition of the proposed microfluidic RAC chip. As shown, the microfluidic RAC chip consists of a PET cover layer (thickness of 0.1 mm) to seal the chambers and channels; a PMMA middle layer (thickness of 3 mm) containing the syringe pump chambers, reagent chamber, sample chamber, serpentine microchannel, and detection chamber; an overflow area; and a PMMA bottom layer (thickness of 1.5 mm) incorporating a narrow microchannel connecting the serpentine microchannel to the detection chamber. The microfluidic RAC chip was designed using CorelDRAW X8 software and manufactured through a CO_2_ laser system (Giant Tech. Co., Ltd., Taipei, Taiwan) [49].

Figure 1b shows that the device incorporated three small circular chambers, each with a diameter of 3.8 mm, to serve as buffers between the syringe pump tubes and the microchip channels to enable precise control of the fluid delivery within the device. The AuNP and pH buffer chambers had diameters of 6 mm, while the sample chamber had a diameter of 10 mm and was connected to a serpentine micro-mixing channel with a width of 0.1 mm. To enhance the absorbance-detection performance, the detection zone was designed with relatively large dimensions of 20 × 2 × 3 mm^3^.

### 2.2. Experimental Process and Smart Analysis Device

Equal volumes (80 μL) of RAC sample solution, modified AuNPs, and pH buffer were injected into the microfluidic RAC chip. The chip was then sealed using the PET film. After the sample was injected during the detection process, sequential control of the syringe pump pushed the pH buffer and AuNP solution into the reaction zone for 60 s. Then, the injection pump was used to drive the reactants through the serpentine mixing channel to ensure better contact and achieve improved mixing effects. After passing through the mixing channel, the reactant flowed into the detection chamber via a narrow channel embedded in the lower substrate, which was designed to inhibit the formation of air bubbles in the detection region. Once the detection region was filled, the microfluidic RAC chip was subsequently inserted into the smart analysis device for absorbance measurement. The overall RAC detection time only takes 3 min to obtain the analysis results.

**Figure 2:**
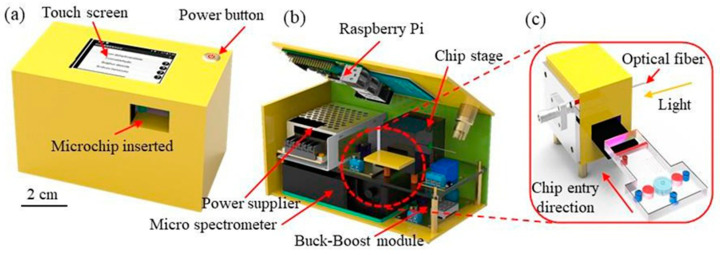
(**a**,**b**) Main components of smart analysis device and (**c**) structure of spectrum detection device.

As shown in Figure 2a,b, the smart analysis device comprised a touch screen (MYW-20429, Meiyagroup, Hsinchu, Taiwan), a power supply (LRS-35-15RS, Meanwell Enterprises Co., New Taipei City, Taiwan), a relay, a microspectrometer (VLS-1000, RAINBOW LIGHT, Taipei, Taiwan), a Raspberry Pi computer (Model B, Raspberry Pi Foundation, Hsinchu, Taiwan), a voltage controller (DC-DC, MP1584, Tainan, Taiwan), and a detection stage. To minimize light interference, the system incorporated light-avoiding measures. In particular, the optical signals were transmitted through an optical fiber to the micro-spectrometer for detection, and the UV-vis spectra were relayed to the Raspberry Pi for analysis via a signal line, as illustrated in Figure 2c.

### 2.3. Preparation of Reagent and Standard Solution

A stock solution was prepared by dissolving 3.3 mg of RAC hydrochloride in 10 mL of deionized (DI) water, resulting in an RAC concentration of 294 ppm. Serial dilutions were then performed to obtain RAC samples with concentrations in the range of 1–100 ppb. Functionalized AuNPs were synthesized using a modified version of the sodium borohydride reduction method [50]. Next, 30 mL of 2 mM HAuCl_4_ was mixed with 170 mL of DI water, and then 17 mL of 20 mM NaBH_4_ was injected into the solution. The formation of AuNPs was confirmed by observing the color change from light yellow to wine red. Subsequently, a mixture of 12 mL of 7 mM glutamic acid and 12 mL of 0.001 wt.% polyethyleneimine was added to the solution. A 10 mM citrate/sodium citrate buffer solution at pH 4 was prepared by dissolving 1.80 g of citric acid and 0.43 g of sodium citrate in 100 mL of DI water.

### 2.4. Sample Pretreatment

To determine the concentration of RAC in real meat samples, the samples were pretreated according to the method issued by the Taiwan Food and Drug Administration (TFDA) (No. 1101901019) [51]. First, ractopamine solutions of different concentrations were cut into meat chunks, and the samples were then homogenized. A 2.0 g aliquot of the homogenized sample was mixed with 20 μL of internal standard solution and 15 mL of 0.2 M sodium acetate buffer solution. The mixture was shaken and extracted at 1000 rpm for 10 min using a high-speed disperser. Subsequently, 100 μL of β-glucuronidase solution was added.

The mixture was hydrolyzed in a 37 °C water bath for 1 h, after which 2 mL of hydrochloric acid was added and shaken again for 10 min. The sample was centrifuged at 10,000× *g* at 4 °C for 10 min, and the supernatant was collected. This supernatant was further centrifuged at 5000× *g* at 4 °C for another 10 min, and the resulting supernatant was used for purification. Purification was performed by passing the supernatant through a solid-phase extraction (SPE) column to collect the eluate. The eluate was evaporated to dryness under nitrogen at 65 °C.

The residue was dissolved in 1 mL of a 5 mM ammonium acetate–methanol solution (9:1, *v*/*v*), mixed thoroughly, and centrifuged at 5000× *g* for 5 min. The supernatant was collected as the original test solution. An aliquot of 500 μL of the original test solution was taken, and the volume was adjusted to 1000 μL with the 5 mM ammonium acetate–methanol solution (9:1, *v*/*v*) to serve as the analytical sample.

## 3. Results and Discussion

### 3.1. Optimization of Operating Conditions

**Figure 3:**
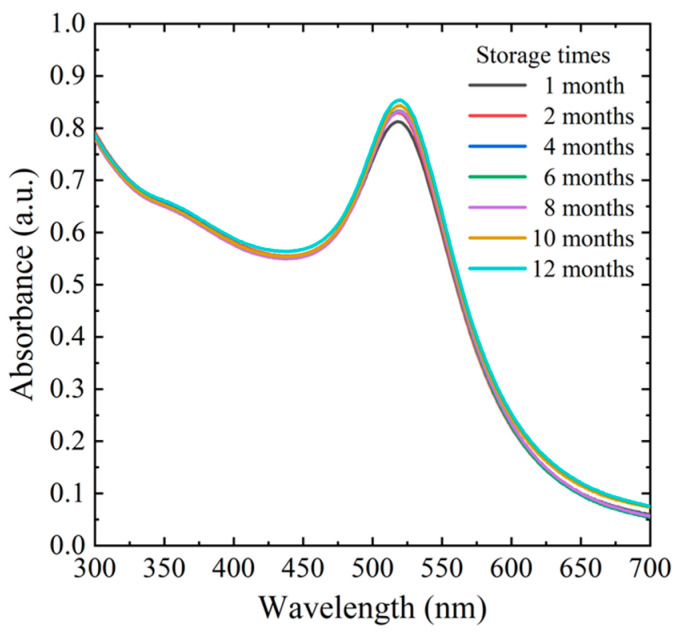
Surface plasmon resonance absorption of GLU-PEI-AuNPs after different storage times.

Owing to the electrostatic repulsion force generated by the negatively charged surfaces of the GLU-PEI-AuNPs, the AuNPs may separate in solution [48]. Thus, to ensure their stability and maintain their characteristic wine-red appearance, the newly synthesized GLU-PEI-AuNPs were covered with aluminum foil to shield them from light and then stored at 4 °C until required for use. Figure 3 shows the UV-vis spectra of GLU-PEI-AuNP samples stored for various time intervals between 1 and 12 months. All the samples show a strong surface plasmon resonance absorption peak at 518 nm, irrespective of the storage duration. Thus, the stability of the synthesized GLU-PEI-AuNPs under the adopted storage conditions is confirmed.

In the reagent, the GLU and PEI compounds serve distinct functions. GLU captures the -OH groups on the RAC through its -C=O groups via hydrogen bonding, while PEI modifies the surface charge of the AuNPs, thereby suppressing electrostatic repulsion and ensuring a stable dispersion. However, both GLU and PEI bind to the AuNPs through their -NH groups. In the proposed chip, the capture of RAC occurs predominantly during mixing in the serpentine channel, and the concentrations of GLU and PEI significantly affect the resulting reaction. Thus, an appropriate specification of the GLU and PEI concentrations is essential for optimizing the detection performance.

**Figure 4:**
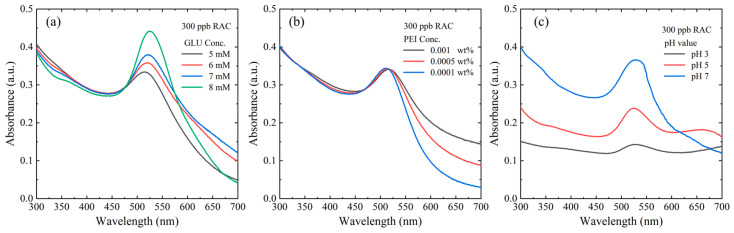
(**a**) Absorbance of reactants with different GLU concentrations and 300 ppb RAC, (**b**) absorbance of reactants with different PEI concentrations and 300 ppb RAC, and (**c**) absorbance of reactants with 300 ppb RAC and different pH values.

Figure 4a shows the UV-vis spectra of GLU solutions with concentrations of 5, 6, 7, and 8 mM mixed with 300 ppb RAC at a PEI concentration of 0.0001 wt.% and a pH of 7. As the GLU concentration increases, the peak intensity shifts to the right, indicating a stronger binding of the RAC to the GLU and a corresponding increase in the AuNP size. The intensity spectrum of the sample with a GLU concentration of 8 mM is clearly distinguishable from the spectra of the other samples and indicates a color shift from red to purple. Thus, to ensure consistency in the colorimetric response of the AuNPs, an 8 mM GLU concentration was discounted. Among the remaining samples, the sample prepared using a GLU concentration of 7 mM shows the highest UV-vis intensity. In other words, a concentration of 7 mM results in an excellent binding ability between the GLU and the AuNPs, while maintaining the AuNP color in the red range and inducing a noticeable wavelength shift. Thus, a GLU concentration of 7 mM was used in all the remaining experiments.

Figure 4b shows the UV-vis spectra of PEI solutions with concentrations of 0.0001, 0.0005, and 0.001 wt.% mixed with 300 ppb RAC at a GLU concentration of 7 mM and a pH of 7. For wavelengths greater than 518 nm, the intensity increases significantly with an increasing PEI concentration. In other words, for a constant GLU concentration of 7 mM, the binding of the PEI to the AuNPs, and the corresponding AuNP particle size, increases as the PEI concentration increases. Consequently, the optimal PEI concentration was determined to be 0.001 wt.%.

The pH of the buffer solution also has a significant effect on the reaction outcome. Figure 4c shows the UV-vis spectra of 300 ppb RAC solutions with GLU and PEI concentrations of 7 mM and 0.001 wt.%, respectively, and pH values of 3, 5, and 7. A prominent peak is again observed at a wavelength of 518 nm. For pH values of 5 and 7, the intensity reduces as the wavelength increases. By contrast, for the sample with a pH of 3, the intensity increases as the wavelength increases beyond 600 nm owing to the formation of zwitterionic GLU, which promotes hydrogen bonding between the GLU-PEI-AuNPs and the RAC [52]. Moreover, two distinct peaks are observed at 518 nm and 693 nm. Overall, the results confirm that pH 3 represents the optimal condition for RAC capture.

### 3.2. Calibration of Microfluidic Detection System

**Figure 5:**
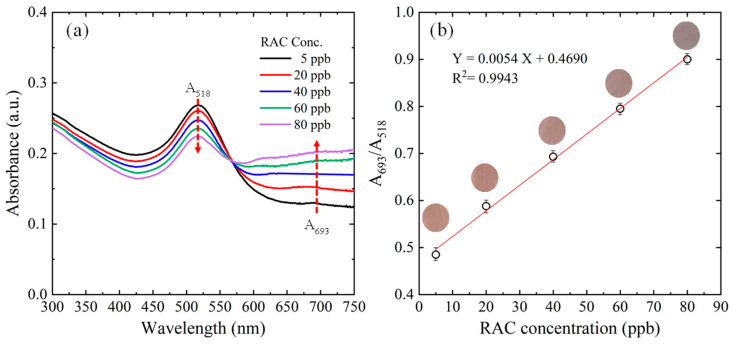
(**a**) UV-vis spectra of reactants with RAC concentrations ranging from 5 to 80 ppb and (**b**) linear regression results for variation in absorbance ratio with RAC concentration.

Under the optimal reaction conditions presented in Section 3.1, absorbance values were measured for five control samples with known RAC concentrations ranging from 5 to 80 ppb using a microfluidic detection platform. Figure 5a presents the measured UV-vis spectra over the wavelength range of 300–800 nm. For each sample, a prominent peak is observed at 518 nm and a low-grade peak appears at 693 nm. Figure 5b shows that the absorbance ratio (A_693nm_/A_518nm_) exhibits a strong linear relationship with RAC concentration over the considered range. Detailed analysis shows that the relationship between the A_693nm_/A_518nm_ ratio (Y) and RAC concentration (X) is Y = 0.0054X + 0.4690, and the correlation coefficient is R^2^ = 0.9943.

The limit of detection (LOD) of the proposed platform was defined as $\mathrm{LOD}=\frac{3\times \sigma }{S}$, where *σ* is the standard deviation of the blank and *S* is the slope of the standard deviation of readouts for five blank samples. From this relation, the LOD of the proposed platform was obtained as 1.0 ppb.

### 3.3. Practical RAC Measurement Performance of Microfluidic System

**Figure 6:**
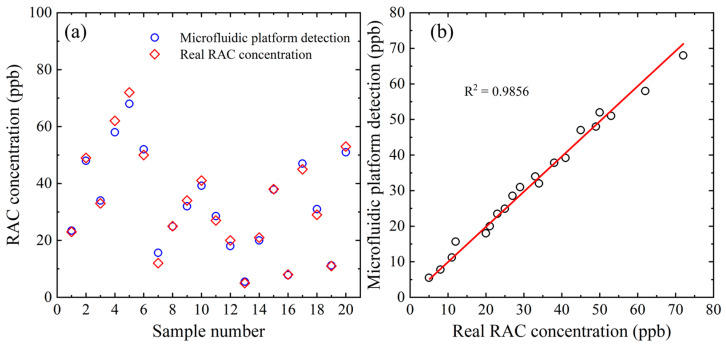
(**a**) Comparison of RAC concentration results obtained using proposed microfluidic chip and true RAC concentrations. (**b**) Correlation between RAC detection results acquired using proposed microfluidic RAC chip and true RAC concentrations.

The feasibility and accuracy of the proposed microfluidic platform were assessed by measuring the absorbance values of 20 samples with unknown RAC concentrations in the range of 1~100 ppb and then converting these absorbance values into the corresponding RAC concentrations using the calibration equation given above. The scatter and linear regression plots presented in Figure 6a,b, respectively, show a good agreement between the two sets of results. The recovery rate was 91.2% to 107.9%, and the linear correlation coefficient between the measured and actual concentrations was R^2^ = 0.9856.

The reliability of the current microfluidic detection platform was examined by analyzing the RAC concentrations of 50 commercial meat samples. Sample preprocessing was performed using the 1101901019 standard methods published by the Taiwan Food and Drug Administration (TFDA). First, 2 g of the sample was dissolved in 15 mL of 0.2 M sodium acetate buffer and stirred with a high-speed disperser for 10 min. Next, 100 µL of β-glucuronidase solution was added and hydrolyzed in a 37 °C water bath for 1 h. Two milliliters of hydrochloric acid was added, stirred for 10 min, and centrifuged at 10,000 rpm for 10 min at 4 °C. The supernatant was collected and rinsed with 12 mL of methanol water (95:5, *v*/*v*) solution. Next, 1 mL of 5 mM ammonium acetate (9:1, *v*/*v*) solution was and centrifuged at 5000 rpm for 5 min. Five hundred microliters of the supernatant was taken as the initial measurement solution, to which 5 mM ammonium acetate (9:1, *v*/*v*) solution was added for a total volume of 1000 µL to prepare the measurement solution. The RAC concentration of each sample under optimal reaction conditions was determined using the calibration formula outlined in Section 3.2. The RAC concentrations were also detected using a standard LC-MS/MS technique by the Technical Service Center of the National Animal Industry Foundation (NAIF) in Taiwan using the same standard method [51].

Among the 50 meat samples, the RAC concentrations of 40 samples were too low to be detected using either method. Thus, only ten sets of comparative measurements were obtained, as shown in Table 1. The accuracy of the proposed platform was defined as $Accuracy\;\left(\%\right)=(1-\left|\frac{proposed\;method-official\;method}{official\;method}\right|)\times 100$. The accuracy varied between 91.67% (Sample #9) and 97.90% (Sample #3). In other words, the accuracy of the proposed platform is better than 8.33%, which also demonstrates the platform’s excellent specificity and selectivity in identifying target analytes. Table 2 compares the proposed microfluidic RAC detection platform with various other methods reported in the literature for RAC detection in foods.

## 4. Conclusions

This study presents a simple microfluidic platform to measure RAC concentration in food products. The platform consists of a low-cost PMMA/PET microfluidic RAC chip and a self-built micro-spectrophotometer smart analysis device. In the detection process, 80 μL each of RAC sample, GLU-PEI-modified AuNPs, and citrate/sodium citrate buffer were injected into the microfluidic chip using syringe pumps. The reactants were then driven through a serpentine mixing channel by a finger pump and into a rectangular detection chamber. The RAC chip was then transferred to the micro-spectrophotometer smart analysis device, where the ratio of the absorbance at wavelengths of 693 nm and 518 nm was used to inversely derive the RAC concentration using a pre-prepared calibration equation.

The reliability and applicability of the proposed microfluidic detection platform was demonstrated by analyzing the RAC concentration in 50 commercial meat samples. The measured RAC concentration values had been demonstrated to deviate from those obtained using a conventional macroscale LC-MS/MS method by no more than 8.33%. Thus, the platform provides a low-cost, reliable, and accurate method for determining the RAC concentration in meat and other livestock products without the need for bulky and expensive analytical apparatus.

## Figures and Tables

**Table 1:** The RAC concentration detection results of 10 commercially available meat samples were obtained using the current microfluidic detection platform and conventional LC-MS/MS method. (Please note that each sample was measured in five replicates to obtain the RAC value.).

NO.	Sample	Microfluidic Detection (ppb)	NAIF Analysis (ppb)	Error (%)
1	Muscle 1 (Cattle)	112.5	118	4.89
2	Muscle 2 (Cattle)	3.2	3	6.67
3	Liver (Cattle)	58.2	57	2.10
4	Kidney (Cattle)	102.2	97	5.36
5	Fat (Cattle)	51.8	54	4.07
6	Muscle 1 (Porcine)	8.3	8	3.75
7	Liver (Porcine)	40.5	39	3.85
8	Kidney (Porcine)	14.6	15	2.67
9	Fat (Porcine)	13.0	12	8.33
10	Muscle 2 (Porcine)	N.D.	N.D.	-

N.D.: Non-Detectable.

**Table 2:** Qualitative comparison of proposed microfluidic platform detection system with other methods reported in the literature for RAC detection in food.

Method	Sample Type	Detection Method	Sample Consumption	Analysis Time	Detection Range (ppb)	Price	Instrument Type	LOD (ppb)	Recovery (%)	Ref.
GC-MS	Feed	GC	10 mL	26 min	8.9~452.1	High	Benchtop	3.6	74.5~91.2	[52]
MWCN Modified Sensor	Pork	EC	5 mL	3 min	44.6~1800	High	Benchtop	17.8	93.1~107.2	[53]
C_3_N_4_/Cu@ CoO/NC sensor	Pork	EC	5 mL	3 min	0.005~32.73 μmol/L	High	Benchtop	1.53 mmol/L	96.5~102.2	[9]
Sulfanilic Acid Modified Nanoparticles	Pork	Colorimetric	50 mL	18 min	4.5~31.6	Low	Benchtop	1.5	94.4~112.5	[54]
Gold Nanoparticles	Pork urine	Colorimetric	0.01 mL	5 min	37.1~334.5	Low	Benchtop	12.4	98~104.4	[55]
LLE and LLE-SPE	Pork Urine	SERS	1 mL	<1 min	N.D.	High	Benchtop	355.6	N.D.	[56]
UPLC-ID-MS/MS	Mutton	MS/MS	0.2 mL	<5 min	1.0~15.0	High	Benchtop	0.01	99.0~99.9	[13]
Immuno-assay	Meat	Glucometer	0.2 mL	<10 min	0.038~5.0	High	Handheld	0.0158	79.4~106.8	[17]
Current Platform	Meat	Spectrum	0.08 mL	3 min	5~80	Low	Handheld	1.0	91.2~107.9	This work

EC: Electrochemical; GC: Gas chromatography; GC-MS: Gas chromatography–mass spectrometry; LLE: Liquid–liquid extraction; LLE-SPE: Liquid–liquid extraction and solid-phase extraction; MWCN: Multi-walled carbon nanotube; SERS: Surface-enhanced Raman spectroscopy; UPLC-ID-MS/MS: Ultra-performance liquid chromatography tandem isotope dilution mass spectrometry.

## Data Availability

The data presented in this study are available upon request from the corresponding author.
